# Mass stranding of common (weedy) seadragons (*Phyllopteryx taeniolatus*) in Sydney: impacts and implications

**DOI:** 10.1111/jfb.70019

**Published:** 2025-03-13

**Authors:** David J. Booth, Andrew Trevor‐Jones, Selma Klanten, Giglia A. Beretta

**Affiliations:** ^1^ Fish Ecology Lab, School of Life Sciences, University of Technology Sydney Sydney New South Wales Australia

**Keywords:** citizen science, climate‐change storms, seadragon strandings

## Abstract

In April 2022, mass stranding of weedy (common) seadragons occurred, with a total of over 200 individuals washed ashore on beaches in the Sydney, Australia region, recorded by citizens. Causes of the stranding, which is unprecedented, were likely related to a series of east coast low storm events, leading to record wave heights, record coastal rainfall, and potential loss of critical food sources (schooling mysid crustaceans). A significant proportion of the local population was likely lost in this series of events, indicating a future threat to seadragons, with east coast low intensity predicted to increase under human‐caused climate change.

The Syngnathidae, including seadragons (see Figure 1a), seahorses, pipehorses and pipefishes, are a family of morphologically diverse fishes characterized by small home ranges, sparse distributions and low fecundity (Foster & Vincent, 2004; Sanchez‐Camara et al., 2005; Sanchez‐Camara & Booth, 2004). They are cryptic fish typically associated with structurally complex habitats, such as kelp, seagrass, corals and sponges (Harasti et al., 2014; Kendrick & Hyndes, 2003; Sanchez‐Camara et al., 2005). These habitats support an abundance of food for syngnathids, for example small crustaceans (Foster & Vincent, 2004; Manning et al., 2019), and allow for effective predator avoidance (Curtis & Vincent, 2005). Seadragons (weedy [*P. taeniolatus*], leafy [*Phycodurus eques*], ruby [*Phyllopteryx dewysea*]) are the largest members (adults range 35–45 cm length) of the Family Syngnathidae, and all three seadragon species are endemic to southern Australia, living in close association with kelp or seagrass beds on or adjacent to shallow rocky reefs, 2–30 m depth (Browne et al., 2008). Seadragons are highly philopatric, with limited dispersal (Sanchez‐Camara & Booth, 2004), so local disturbances may have a disproportionate effect on spatial structuring, and loss of seadragons can lead to slow recovery (see Pollon, 2017a, 2017b).

**FIGURE 1:**
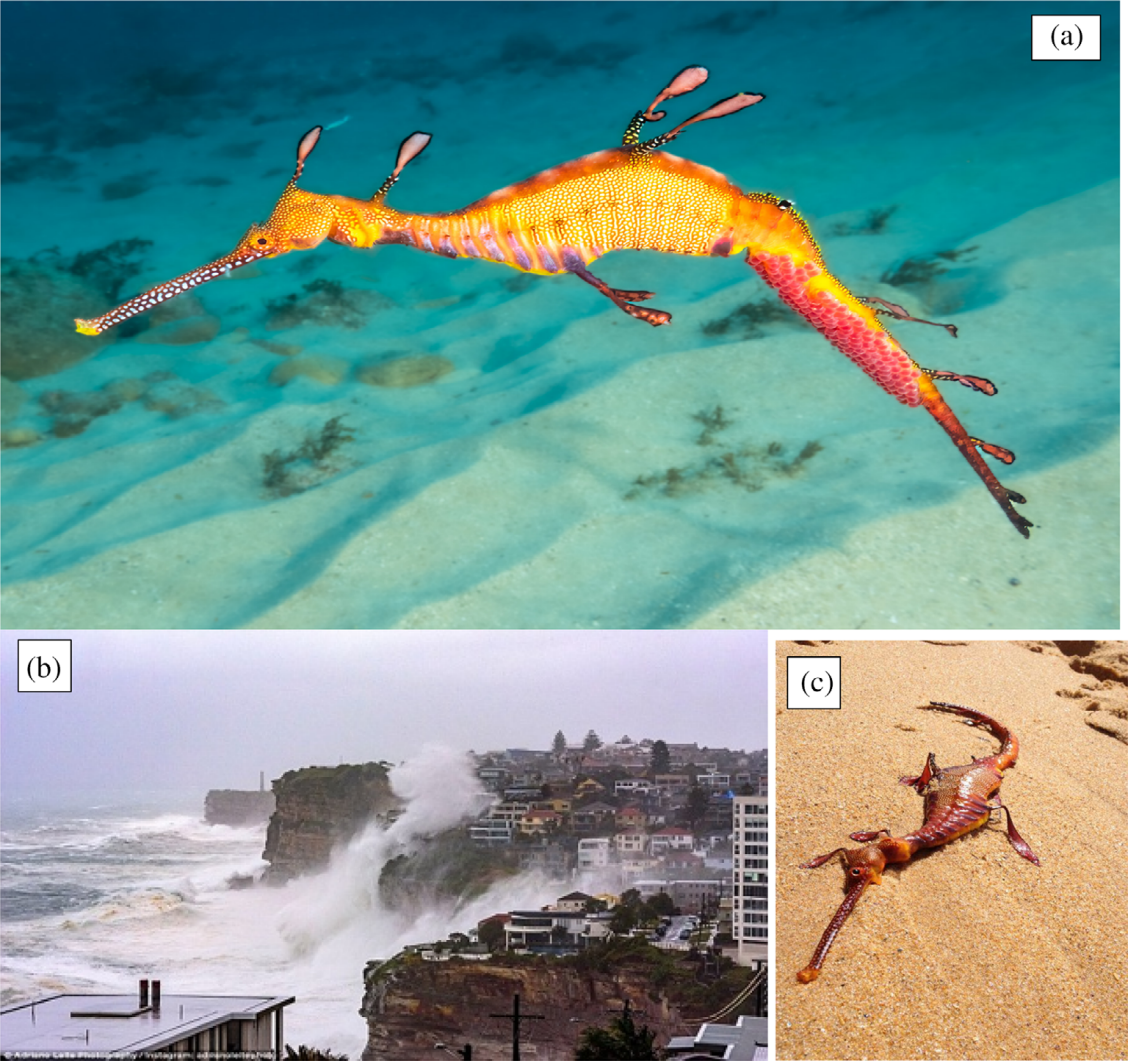
(a) A weedy seadragon in Sydney with an egg mas on its tail (photograph: D. Harasti). (b) Sydney coastline under a severe storm. (c) Stranded seadragon carcass on a Sydney beach (photograph: M. Bordieri, 6 April 2022).

Here we describe a mass stranding event for weedy (common) seadragons (*Phyllopteryx taeniolatus* [Syngnathidae] Lacepede, 1804), and we discuss its potential causes and consequences for local populations. Weedy seadragons are found only on the temperate reefs of southern Australia, ranging from Port Stephens, New South Wales (latitude −32.6928, longitude 152.1210) to Geraldton, Western Australia (latitude 28.77897, longitude 114.61459) (Edgar, 2008; Sanchez‐Camara et al., 2011).

During summer 2022, south‐eastern Australia was subjected to a marine heat wave (Oliver et al., 2018) which drove late summer (February) water temperatures up to 26°C in Sydney (2–4°C above normal). This was accompanied by a series of severe “East Coast low” storms that battered the coast (Figure 1b). People walking the beaches around Sydney reported (via email, phone, online platforms such as i‐Naturalist) that large numbers of carcasses of weedy seadragons had washed ashore, and under DPI permit (FP22/21) we coordinated data and specimen collection from March 2022 until the present.

We collected carcasses and associated data (body length, condition), plus images (location and date‐linked) of beached dead weedy seadragons provided by citizens (e.g. Figure 1c), and added these data to long‐term data we have collected on seadragon strandings in this region. Sex was determined from images or from dead specimens (females have a deeper abdominal area; Klanten et al., 2020). If separately reported washup images were in close proximity, with no specimen collected, we took particular care to ensure images were not of the same individual (we did discard several “doubles”), and based this on any markings, exact location (e.g. next to physical feature) and the shape of the specimen (e.g. stiffened and bent in a certain orientation). We feel that this avoided any double counts. Overall, we collected 63 carcasses and 218 images of beached dead weedy seadragons. Samples were spread along 120 km of shoreline from New South Wales Central Coast (Gosford) to Wollongong (40 km South of Sydney), and four regions (Central Coast, Sydney North, Sydney South, Wollongong) were analysed separately, with only Sydney South showing low washup frequency (Figure 2). Seadragon stranding weekly frequency is shown in Figure 2a, and indicates that March and April 2022 had the highest numbers of strandings, with over 80 in the week of 4 April. At Sydney North, with a very exposed coast and high washup frequency, reported sightings of weedy seadragons underwater dropped to near zero after April 2022, and were still rare in June 2024 (pers. obs.). Sydney South showed the lowest wash‐up frequency, with field sites on the south shore of Botany Bay protected from prevailing winds, and despite a dip in numbers in March–April 2022, seadragon sightings in this area increased (Figure 2c). A similar number of males and females were stranded overall (Chi Squared 0.47 *p* > 0.05, *n* = 63).

**FIGURE 2:**
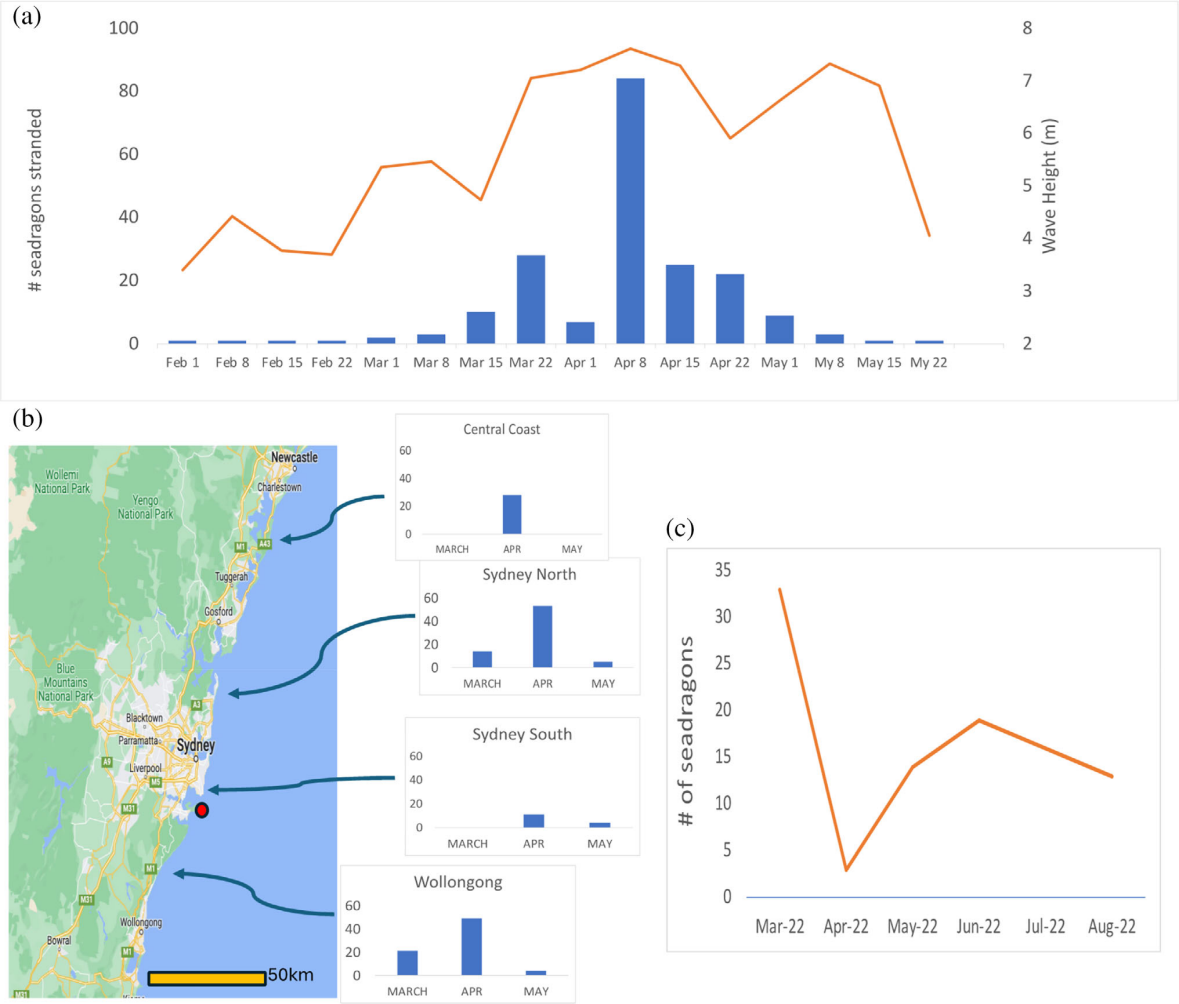
Data on seadragon strandings in Sydney region. (a) Total strandings by week, February–May 2022 (bars). Sydney offshore maximum waver heights February–May 2022 (Manly Hydraulics Lab NSW Coastal Wave dataset) (line). (b) Spatial/temporal strandings in Sydney region, 2022 (# stranded). The red circle is Kurnell. Botany Bay. (c) Live seadragons sighted in Kurnell, Botany Bay Sydney 2022 (A. Trevor‐Jones, unpub. data).

Marine fish strandings are widespread and can involve many environmental and physiological causes (e.g. Brusius et al., 2022). Causes of the weedy seadragon strandings seem to be related to the summer marine heatwave or the series of severe east coast low storm events in 2022 in the Sydney region. First, record wave heights with associated surge were recorded in Sydney in April 2022 (over 14 m inshore: e.g. Dowdy et al., 2019) and data records from 1 February to 31 May 2022 indicate significant wave events in March to April, coinciding with the April stranding event (Figure 2a). In addition, 2022 received record rainfall in this region (over 2500 mm for 2022, more than double the long‐term average; Bureau of Meteorology http://www.bom.gov.au/climate/current/annual/nsw/archive/2022.sydney.shtml), with the period January–April receiving over 1600 mm compared to a decade average over that period of 430 mm (DJ Booth, unpub. data). Finally, mysid crustacean swarms, a major food source for seadragons (e.g. Allan et al., 2022a, 2022b) disappeared from Sydney coastal waters from January to May 2022 (regular mysid collection data; SeaLife Aquarium Sydney; T. Burd, pers. obs.; D Booth, pers. obs.) and so starvation may have occurred.

Seadragons perform poorly under surge waves and are subject to barotrauma (e.g. https://divernet.com/scuba-diving/taking-care-in-the-dragons-lair/). Record wave heights (over 14 m) were reported off the Sydney coast at that time, suggesting large pressure changes in coastal waters. While subsequent large wave events have been recorded, these have not resulted in significant seadragon stranding events, possibly because they have not been accompanied by such severe rainfall and loss of mysid shrimp populations, and low‐density populations may recover very slowly.

There is concern that the large number of seadragon strandings, following a series of severe storms and flooding, has likely led to local population declines. Longshore surveys using underwater scooters along the 100 km of coastline are currently underway (M. Bordieri, unpub. data), with total abundance estimated at less than 1000 individuals. Unpublished data from the 1990s to the present indicate that washups are normally rare, none to one per month (e.g. Janine Baker, DragonSearch, unpub. data). Seadragon young do not disperse more than a few kilometres, and most tagged adults move under 200 m over their lifetime of over 8 years (Sanchez‐Camara et al., 2011). Therefore, they have high site fidelity, which is also linked to strong genetic structure across their range (Klanten et al., 2020; Stiller et al., 2023). This is uncommon for marine fishes, which usually have wider dispersal (Baetscher et al., 2019). The low dispersal capacity and structured populations of such species means that local losses of individuals in one part of their range, as documented here, can lead to loss of genes/alleles and local extinctions (e.g. Weiss et al., 2022). Marine heatwaves and climate‐related storms are predicted to increase in intensity globally, including in southeast Australia, with increased rainfall and winds predicted under climate change (Cavicchia et al., 2020) so they could be a major future risk to the viability of seadragons in parts of their geographic range.

Charismatic species such as the weedy seadragon can be used as effective flagship species in conservation, which in turn benefits numerous other marine species and habitats (Clucas et al., 2008). Whilst the species is currently listed as ‘Least Concern’ on the IUCN Red List (https://www.iucnredlist.org/species/17177/67624517), there is evidence that the species has declined in abundance at numerous sites in eastern Australia (Edgar et al., 2023; Sanchez‐Camara et al., 2011), which is especially worrying given their spatial structuring. Finally, the value of citizen science in observing the impacts of climate change and extreme events is recognized (Kelly et al., 2020). In Australia, a number of citizen science programs have been established, including for sea dragons (e.g. SeadragonSearch, https://seadragonsearch.org/). The large network we have developed following the 2022 strandings will have value in future detections of marine organism strandings.

## AUTHOR CONTRIBUTIONS

Conceptualisation: D Booth, S Klanten, G Beretta; Conduct research and data collection: D Booth, S Klanten, G Beretta, A Trevor‐Jones. Manuscript preparation and editing: D Booth, S Klanten, G Beretta, A Trevor‐Jones
